# Reclaimed and Up‐Cycled Cathodes for Lithium‐Ion Batteries

**DOI:** 10.1002/gch2.202200046

**Published:** 2022-06-09

**Authors:** Dominika Gastol, Jean Marshall, Elizabeth Cooper, Claire Mitchell, David Burnett, Tengfei Song, Roberto Sommerville, Bethany Middleton, Mickey Crozier, Robert Smith, Sam Haig, Con Robert McElroy, Nick van Dijk, Paul Croft, Vannessa Goodship, Emma Kendrick

**Affiliations:** ^1^ School of Metallurgy and Materials University of Birmingham Birmingham B15 2TT UK; ^2^ WMG University of Warwick Coventry CV4 7AL UK; ^3^ ICoNiChem Widnes Ltd Moss Bank Road Widnes Cheshire WA8 0RU UK; ^4^ TFP Hydrogen Products Units 5 & 6 Merchants Quay Pennygillam Industrial Estate Launceston Cornwall PL15 7QA UK; ^5^ MSolv Oxonian Park Langford Locks Kidlington Oxford OX5 1FP UK; ^6^ RSBruce Metals and Machinery Ltd March Street, Sheffield South Yorkshire S9 5DQ UK; ^7^ Green Chemistry Centre of Excellence Department of Chemistry University of York Heslington York YO10 5DD UK

**Keywords:** disassembly, Li‐ion batteries, reclamation, shredding, up‐cycled cathode

## Abstract

As electric vehicles become more widely used, there is a higher demand for lithium‐ion batteries (LIBs) and hence a greater incentive to find better ways to recycle these at their end‐of‐life (EOL). This work focuses on the process of reclamation and re‐use of cathode material from LIBs. Black mass containing mixed LiMn_2_O_4_ and Ni_0.8_Co_0.15_Al_0.05_O_2_ from a Nissan Leaf pouch cell are recovered via two different recycling routes, shredding or disassembly. The waste material stream purity is compared for both processes, less aluminium and copper impurities are present in the disassembled waste stream. The reclaimed black mass is further treated to reclaim the transition metals in a salt solution, Ni, Mn, Co ratios are adjusted in order to synthesize an upcycled cathode, LiNi_0.6_Mn_0.2_Co_0.2_O_2_ via a co‐precipitation method. The two reclamation processes (disassembly and shredding) are evaluated based on the purity of the reclaimed material, the performance of the remanufactured cell, and the energy required for the complete process. The electrochemical performance of recycled material is comparable to that of as‐manufactured cathode material, indicating no detrimental effect of purified recycled transition metal content. This research represents an important step toward scalable approaches to the recycling of EOL cathode material in LIBs.

## Graphical abstract

Black mass containing mixed LiMn_2_O_4_ and Ni_0.8_Co_0.15_Al_0.05_O_2_ from a Nissan Leaf pouch cell is recovered via two different recycling routes, shredding or disassembly. The reclaimed material is further treated to reclaim the transition metals salts. Upcycled LiNi_0.6_Mn_0.2_Co_0.2_O_2_ (NMC 622) is produced. The energy inputs of the processing, purity, and performance of the NMC 622 from the two processes are evaluated.

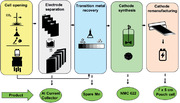

## 1 Introduction

Lithium‐ion batteries (LIBs) have undergone extensive development since their invention in the 1980s,^[^ ^1^, ^2^, ^3^ ^]^ and their commercialization has accelerated as electric vehicles become more widespread. LIBs have a number of commercial advantages. The operating principle of LIBs relies on the intercalation and deintercalation process of Li^+^ between the electrodes. During charging, the positive electrode acts as a source of Li^+^; the power source applied to the battery oxidizes the transition metal oxide, releasing the Li^+^ to the electrolyte and simultaneously releasing electrons into the external circuit. The electrons combine with intercalated Li^+^ at the graphite‐based negative electrode. During discharge the reverse reaction takes place spontaneously, where both electrons and Li^+^ are released from the negative electrode and the current from released electrons can power a load.^[^ ^4^ ^]^

Since LIBs are transportable, relatively lightweight, and can be recharged using renewable energy sources, they have a role to play in humanity's ongoing attempts to decrease our reliance on fossil fuels, particularly in the transport sector. Furthermore, they have a low tendency to self‐discharge and a high energy density; again, these properties make them particularly suitable for applications in transport.^[^ ^5^ ^]^ However, as the number of electric vehicles on the road increases, so does the number of battery packs that are coming to their end‐of‐life (EOL), and this now poses a significant challenge in terms of reuse and recycling of the batteries.

Recycling of batteries is a complex process; this is partly due to the number of different chemical components in a battery,^[^ ^6^ ^]^ and partly due to safety considerations since some of the materials involved are flammable and/or toxic.^[^ ^7^, ^8^ ^]^ Furthermore, batteries that have reached their EOL have different histories and may therefore have slightly different chemical composition.^[^ ^9^, ^10^, ^11^, ^12^, ^13^ ^]^ Despite the difficulty and expense of the recycling procedure, however, it is imperative that recycling of battery components is embraced. This is first because the toxic nature of some battery components makes them a hazard to health and the environment if they are disposed of unsatisfactorily, and second because the manufacture of LIBs relies on some resources that are critical materials (i.e., cobalt, graphite, and lithium)^[^ ^14^ ^]^ and so an effective recycling strategy is required in order to secure supply of these materials and minimize the strain on global resources.^[^ ^15^, ^16^, ^17^ ^]^

Several commercial processes are being established globally to recycle and reclaim useful materials from spent batteries.^[^ ^18^, ^19^ ^]^

Currently, most recycling methods are based around an approach where battery cells are mechanically shredded, before the valuable components are concentrated in subsequent steps. However, all shredding procedures have limitations; they produce mixed waste streams that cannot always be easily purified. Proposed methods for improved separation of the components of shredded material include froth floatation^[^ ^19^, ^20^ ^]^ (which selectively separates materials based on their hydrophobicity), shear de‐agglomeration,^[^ ^21^ ^]^ selective leaching (in which some chemical components preferentially dissolve into solvents before others),^[^ ^22^, ^23^, ^24^, ^25^, ^26^ ^]^ and mechanochemical recovery techniques.^[^ ^27^, ^28^, ^29^ ^]^

Previous work demonstrated that purer waste streams can be achieved if the battery is disassembled rather than shredded.^[^ ^14^, ^30^, ^31^ ^]^ This procedure must be controlled, due to the fact that some of the internal battery components can react with water and oxygen in the atmosphere; in an appropriate environment, the disassembly can be automated and there are examples for different cell types where this has been achieved.^[^ ^32^ ^]^ Some quantitative information has been published on the chemical composition of batteries containing different cathode materials.^[^ ^32^, ^33^ ^]^ Obviously, the overall chemical composition will depend on the cathode chemistry chosen, but there will also be considerable variability between batteries from different manufacturers and between batteries that have undergone different levels of use. An ideal recycling process would be sufficiently robust that it could be used for all batteries, but in practice the exact procedure used will depend on the architecture and chemistry of battery being recycled.

The highest‐value materials in LIBs tend to be the metals found in the cathode (cobalt, nickel, etc.) that account for ≈40% of the total material value in the LIB.

There can be differentiated three battery recycling approaches: pyrometallurgical and hydrometallurgical processes as well as direct recycling approaches.^[^ ^16^, ^34^ ^]^ The first two methods are available on an industrial level, whereas the third one is being tested at the laboratory and pilot plant level.^[^ ^16^ ^]^

Pyrometallurgy is an energy‐intensive smelting process which usually involves two steps: first, LIBs are burned in a smelter, where the components are broken down and the organic materials are burned away.^[^ ^35^ ^]^ Next, new alloys are generated by carbon reduction processes. The metal alloys can be further separated by hydrometallurgical processes into the component metals. In this process, Ni, Co, Cu are being recovered, whereas, the anode, electrolyte and plastics are oxidized to supply energy for the process. Li can be also recovered from the slag by additional processes, whereas Al acts as a reductant in the furnace and reduces need for a fuel. The main advantages of pyrometallurgy are the simplicity of the process and its industrial scale. However, it possesses some major disadvantages, such as: CO_2_ emission, high energy demand for the smelting process, additional processes for recovery of pure metals. In addition to this, with the future trend to produce low Co content EV batteries, the business model will become less attractive.^[^ ^16^ ^]^

The hydrometallurgical approach^[^ ^36^ ^]^ is based on application of aqueous solutions in order to recover targeted metals from the cathode material. It involves chemical processes, leaching, solvent extraction, precipitation, ion exchange, and electrolysis. Three approaches have gained a significant interest, i.e., leaching, solvent extraction, and chemical precipitation.^[^ ^37^ ^]^

Leaching processes can be further subdivided into alkali and acid leaching, where acid leaching results in higher metal leaching efficiency. Acid leaching can incorporate inorganic and organic acid leaching. Organic acid is more favorable as it can reach similar efficiencies with milder environmental impact compared to inorganic acid treatment.^[^ ^37^ ^]^ In addition to this, bioleaching can also offer an environmentally friendly approach, where the acids are generated during microorganisms’ metabolism processes.

Solvent extraction is a process that follows leaching and is aimed at separation of metal ions or impurities between organic solvents and aqueous solutions. As this approach offers high purity products, it is implemented by industry, however, high costs of purification remain as a disadvantage at the moment.

Chemical precipitation relies on separation of metals based on their solubilities at different pHs. Applied precipitants react with transition metal ions and Li^+^ to form insoluble precipitates. Common precipitants are NaOH, H_2_C_2_O_4_, C_4_H_8_N_2_O_2_, H_3_PO_4_, Na_2_CO_3_, Ni, Mn, and Co co‐precipitate as hydroxides that can further be incorporated for cathode fabrication.^[^ ^37^ ^]^

Direct recycling relies on removal of different materials from spent LIBs and reconditioning them. In this approach, cathode mixed‐metal oxides can be incorporated into new cells by maintaining original or incorporating minimal changes to the material crystal morphology. After replenishing the lithium content by hydrothermal or solid‐state reactions, the material can be incorporated into fresh LIB cell.^[^ ^34^ ^]^

Most frequently, a mixture of techniques is employed in order to obtain materials of the desired purity.^[^ ^38^ ^]^

In this work, we explore the effectiveness of different routes to transition metal recovery. For our study, we used waste cells from a Nissan Leaf (Generation 1: LMO (LiMn_2_O_4_)+NCA (Ni_0.8_Co_0.15_Al_0.05_O_2_) cathodes); these were at their EOL state. In this study, we compared the performance of the materials obtained from the shredding route (with and without an applied purification step during hydrometallurgical transition metal recovery) as well as materials obtained via a disassembly process. We analyzed the resulting material and used this recycled component to remanufacture cathode material for the new cathode half‐cells. The energy demand for the utilized recovery processes has been introduced to provide an estimation of the benefits and drawbacks of tested metal recovery and reprocessing routes.

## 2 Results and Discussion

### 2.1 Black Mass Recovery

**Figure 1:**
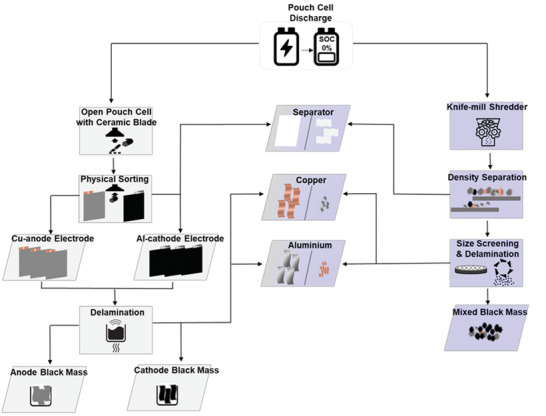
Diagram of disassembly and shredding process applied for cathode black mass recovery.

**Figure 2:**
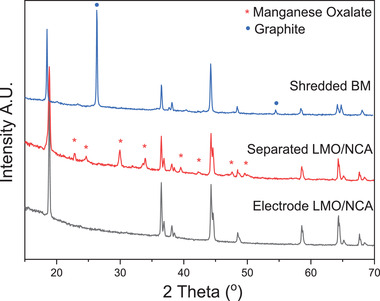
XRD diffraction patterns of the recovered cathodic black mass using oxalic acid bath and ultrasound, and the as‐received electrode from the EOL cell compared with shredded black mass (BM). Active material of LiMn_2_O_4_ and NCA can be observed in the XRD pattern samples for disassembly route, with manganese oxalate present in the reclaimed black mass as a secondary phase, whereas, Ni‐rich cathode material with graphite peaks is observed for shredded BM.

The disassembly of a Nissan module and subsequently pouch cells was performed under a fume hood, **Figure** **1**. A scalpel with a ceramic blade was used to separate the cell's sub‐components (anode, cathode, separator, and pouch) from each other. The remaining pieces of Al and Cu tabs, separators, and pouch were collected separately. It was determined, based on the peeling‐off efficiency, supported with the inductively coupled plasma‐optical emission spectrometry (ICP‐OES) data that the oxalic acid demonstrated the separation efficiency of 98.7% as well as low content of Co, Cu, Mn, and Ni present in the leachate, see Figures S3 and S4 in the Supporting Information, respectively. The composition of the reclaimed cathodic black mass from the disassembly and shredding processes is shown in **Table** **1**. In **Figure** **2**, the X‐ray diffraction (XRD) patterns of the recovered cathodic black mass using oxalic acid have been compared with EOL LMO/NCA electrode and recovered shredded black mass. It can be found that characteristic peaks of the LMO/NCA from the recovered cathodic black mass correspond to the EOL electrode pattern, with some noticeable peaks that can be assigned to the oxalate. Whereas, the black mass from the shredded material demonstrates peaks characteristic to the Ni‐rich cathode material with the presence of graphite contamination.

**Table 1:** ICP‐OES data of the cathode black mass from the shredded and disassembly routes

Element	Shredded material	Disassembled material
C	16.33%	9.14%
Li	2.79%	3.83%
Co	6.06%	1.65%
Ni	11.56%	13.45%
Mn	6.79%	43.78%
Al	4.45%	0.78%
Cu	1.01%	0.06%

It can be noticed that the shredded material contains nearly twice the amount of C that can indicate contamination with an anode black mass; this has been confirmed by XRD data as well, as shown in Figure 2. There is also significantly higher concentration of Al and Cu in the shredded cathode black mass as a result of the contamination from the current collectors. Higher amount of Co in the same fraction can indicate different origin of the cathode material than the EOL LMO/NCA electrodes from disassembly process.

Next, the black mass was then dissolved into solution for further purification, and NMC 622 solutions were made for further cathode manufacture. **Table** **2** shows the resulting levels of transition metal, copper, and aluminium content within these solutions.

**Table 2:** Solutions prepared from the dissolved cathode black masses with a corresponding ICP‐OES elemental content and the final ratios for the NMC 622 synthesis

		Dis	Dis	Shred	Shred	Shred	Shred
Sample no.		D‐54^a)^	D‐97	SP‐65	SP‐50	SU‐63	SU‐51
Black mass (g)		4043.23	4485.13	600	600	850	850
% Recycled		53.76	95.66	64.70	50.30	63.40	50.60
Ni	(ppm, µg g^−1^)	56 480	51 310	56 530	52 440	38 030	38 280
Co	(ppm, µg g^−1^)	18 490	17 280	17 270	16 330	11 830	11 730
Mn	(ppm, µg g^−1^)	17 830	15 680	18 730	17 600	12 900	13 110
Al	(ppm, µg g^−1^)	7	30	27	13	9191	8199
Cu	(ppm, µg g^−1^)	31	15	89	60	2144	1887
Ratio	Ni	6	6	6	6	6	6
	Mn	2.02	1.96	1.96	1.95	1.95	1.97
	Co	1.96	2.01	1.98	1.99	1.99	2

^a)^ D, disassembled, SP is shredded and additionally purified to remove aluminium and copper, and SU is shredded and dissolved with no purification.

**Table 3:** XRD refinement lattice parameters

Sample	*a* [Å]	*c* [Å]	*a*/*c* ratio	Vol [Å^3^]	Lx	Chi2	*R*_p_ [%]	*d*_003_/*d*_104_	*D* [µm]
SU‐51	2.85770	14.24000	0.20068	100.710	1.0	3.11	1.24	1.47	0.88
SU‐63	2.85410	14.22700	0.20061	100.370	1.7	3.54	1.33	1.65	0.52
SP‐50	2.87050	14.22200	0.20184	101.490	3.0	3.00	1.19	1.29	0.29
SP‐65	2.86771	14.21390	0.20175	101.231	3.7	2.98	1.20	1.27	0.25
D‐54	2.86390	14.20400	0.20163	100.890	5.1	2.56	1.10	1.33	0.14
D‐97	2.86480	14.20500	0.20168	100.960	6.4	1.95	1.05	1.19	0.19
STD	2.86765	14.21539	0.20173	101.238	6.6	0.71	1.60	0.95	0.44

### 2.2 Cathode Remanufacture

The solutions prepared from the cathode black mass were utilized directly in the cathode manufacture. The coprecipitation method was employed for the synthesis of Li[Ni_0.6_Mn_0.2_Co_0.2_]O_2_. The mixed transition metal salt solutions were obtained by adjusting the concentration of the reclaimed transition metal content with additional transition metal salts. In some cases, the additional content was from purchased transition metal salts, where in other cases the content of the recycled transition metals was maximized. Two samples were made using precursors manufactured from the cathodic black mass, obtained from the disassembly process. D‐54% and D‐97% contained 54% and 97% of recycled transition metal salt content, respectively. The transition metal salt solutions obtained from the mixed shredded black mass were either further purified (SP‐65%, 50%) or used as‐received (SU‐63%, 51%) with ≈63% and 51% recycled transition metals. A summary of the different precursor salt solutions and their metal content is shown in Table 2.

#### 2.2.1 XRD of Resynthesized Powders

**Figure 3:**
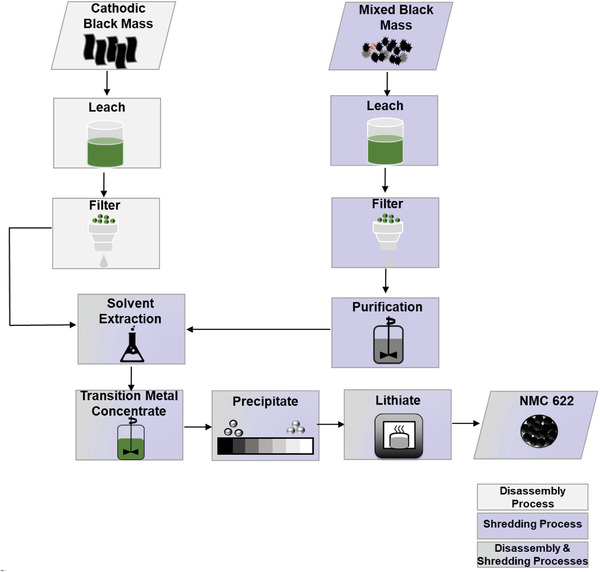
Diagram differentiating processing of the cathode black mass through disassembly route and shredding: purified and unpurified streams.

**Figure** **3**a illustrates the XRD diffraction spectra conducted on the synthesized and commercially available powders.

All patterns have a typical hexagonal structure of α‐NaFeO_2_ with a space group of *R* $\bar{3}$ *mH* (no. 166), peak splitting observed in the [006]/[102] and [108]/[110] doublets also confirms this.^[^ ^39^ ^]^ Sample SP‐65% exhibits the narrowest reflexes that indicate larger mean crystallites. The highest intensity factor of reflexes 003/104 of 1.65 and 1.47 for powders SU‐63% and SU‐51% (from unpurified cathodic black mass) indicates that additional processes applied to the purified materials influence their cation mixing in the lattice. The recycled D‐ 97% material from disassembly stream has a 003/104 ratio of 1.19. There can also be observed some impurity diffraction peaks in the materials: SU‐ 51% and SU‐ 63%. The (**Table** **3**) summarises the lattice parameters obtained from Le Bail refinement analysis.

#### 2.2.2 Scanning Electron Microscopy (SEM) Investigation of the Obtained NMC 622 Active Materials

It can be observed that Li[Ni_0.6_Mn_0.2_Co_0.2_]O_2_ secondary particles of SP‐50%, SU‐51%, SU‐63%, D‐97%, and standard commercial material (STD) exhibit spherical morphology. The pallet‐shaped primary particles were found to be closely aggregated together to form the secondary particles in case of the powders: SU‐51%, SU‐63%, and D‐ 97.0%, respectively. However, the size and the density of the primary particles vary between the investigated materials. As the process of the synthesis was optimized throughout the studies, changed conditions such as stirring rate, pH value, or anion source could have contributed to that phenomena.^[^ ^40^ ^]^ In addition to this, it was reported elsewhere that Cu contamination during the co‐precipitation process can also affect the particles of the precursor.^[^ ^41^ ^]^

**Figure 4:**
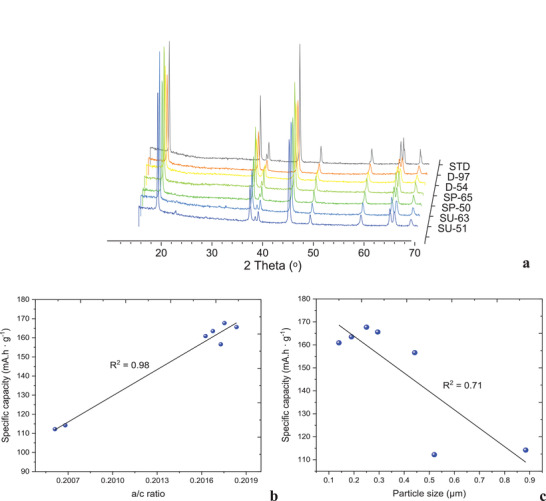
a) XRD diffraction patterns for the synthesized and commercial (STD) NMC 622 powders, respectively. b) Relationship of the specific capacity to the crystal structure size and (c) particle size.

**Figure 5:**
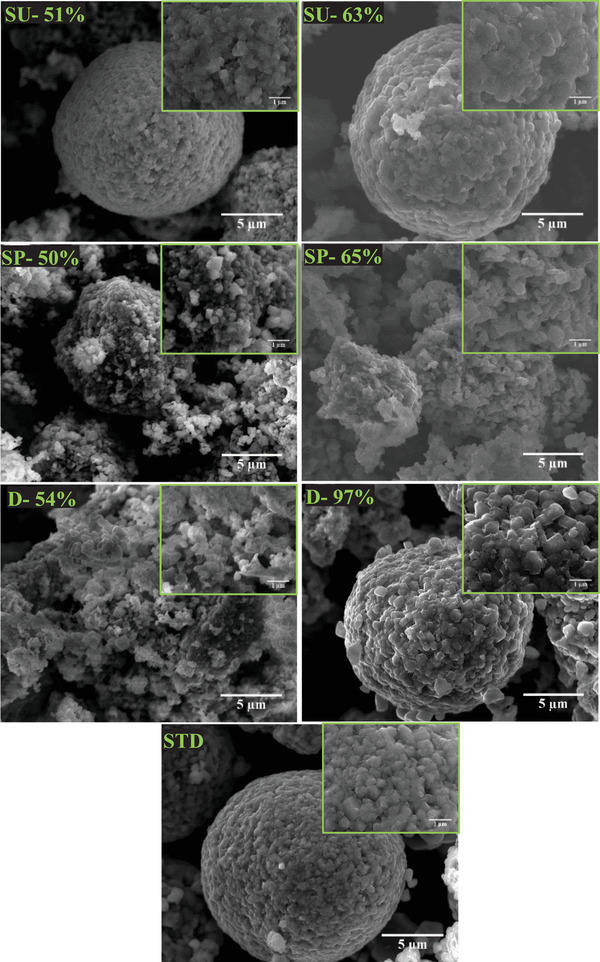
SEM micrographs illustrating morphology of the synthesized NMC 622 powders, obtained from: purified shredded stream (SP): 50%, 65%, shredded unpurified (SU): 51%, 63%, disassembly process (D): 54%, 97%, and standard commercial material (STD), respectively.

D10, 50, and 90 distributions of the primary particles of the synthesized NMC 622 powders have been illustrated in **Figure** **4** based on the image analysis from **Figure** **5**. It can be observed that D90 of the SP‐65% powder exhibits similar particle size to the standard NMC 622. The biggest size of the primary particles was noted for the SU‐63%, whereas, the smallest exhibited D‐54%.

The energy dispersive X‐ray spectroscopy (EDS) analysis of the active materials has been summarized in the graph of Figure S2 in the Supporting Information.

The highest amount of Cu and Al impurities was found in the samples of SU‐51% and SU‐63% that were not purified during the transition metal recovery step. Whereas, no Cu contamination was found in SP‐50%, SP‐65%, and D‐97% samples.

### 2.3 Cathode Remanufacturing and Electrochemical Testing

**Figure 6:**
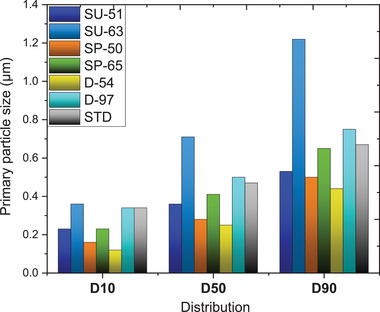
Primary particle size distribution of D10, 50, and 90, respectively, of the synthesized and commercial NMC 622 powders.

**Figure** **6**a presents the capacity values of cathode half‐cells from the formation cycle for all the tested materials. The highest gravimetric capacity of 167.7 mAh g^−1^ with the efficiency of 94.1% was noted for the SP‐65% material obtained from the purified shreddeWd material stream. Whereas, the capacity of 163.5 mAh g^−1^ was demonstrated for material (D‐97%) recovered from the disassembly route with the efficiency only 1.0% lower compared to the best performing recycled material.

It should be highlighted that the cells comprising of NMC 622 material: SU‐63% and SU‐51%—unpurified shredded stream, exhibited ≈50 mAh g^−1^ lower capacity values in comparison to the purified materials. The efficiencies of the first two cycles were noted also to be over 12% lower when compared with the performance of the purified material stream, see **Table** **4**.

**Table 4:** Average measured gravimetric capacities with std. dev. for the tested materials with the corresponding first cycle efficiencies

Material	Gravimetric capacity [mAh g^−1^]	First cycle efficiency [%]
STD	158.0 ± 1.5	89.5 ± 0.5
SP–50%	165.6 ± 0.6	93.1 ± 0.2
SU–51%	114.2 ± 0.3	80.8 ±0.3
D–54%	160.9 ± 0.6	94.2 ± 0.1
SU–63%	112.2 ± 0.5	80.4 ± 0.6
SP–65%	167.7 ± 0.8	94.1 ± 0.2
D–97%	163.5 ± 0.3	93.1 ± 0.1

Figure S2 in the Supporting Information shows the differential capacity for the first two formation cycles of the cathode half‐cells testing the synthesized NMC 622 materials from both recycling streams compared to the standard active material. The cells comprising the electrodes made with SP‐65% and SU‐63% develop polarization, this can be observed on the second cycle. A deformation of the second cycles visible for SP‐50%, SU‐63%, SP‐65%, and D‐97.0% could suggest some processes of material degradation.^[^ ^42^ ^]^

The cell containing SU‐51% demonstrates no characteristic peaks associated with redox reaction of the Ni^+^ and Co^+^.

The previous researchers reported the valences of transition metals, Co and Mn in Ni‐rich NMC materials to be a mixture of Ni ^2+^ and ^3+^, Co^2+^, and Mn^4+^, respectively.^[^ ^39^, ^43^ ^]^ Whereas, it was highlighted elsewhere^[^ ^44^ ^]^ that during the delithiation process Ni is immediately oxidized to Ni^3.7+^ and reduced to Ni^2.9+^ by the end of the lithiation process. Ni does not reach Ni^4+^ due to the upper voltage cut off at 4.2 V. On the other hand, Co is not instantly oxidized, as it remains active at higher voltages.^[^ ^44^ ^]^ It starts to oxidize at 3.8 V, reaching Co^3.5+^. In lithiation, Co is also active in higher voltages and completes the process reaching Co^3+^. Mn^4+^ remains inactive over the voltage window from 2.5 to 4.6 V and NMC composition.

**Figure 7:**
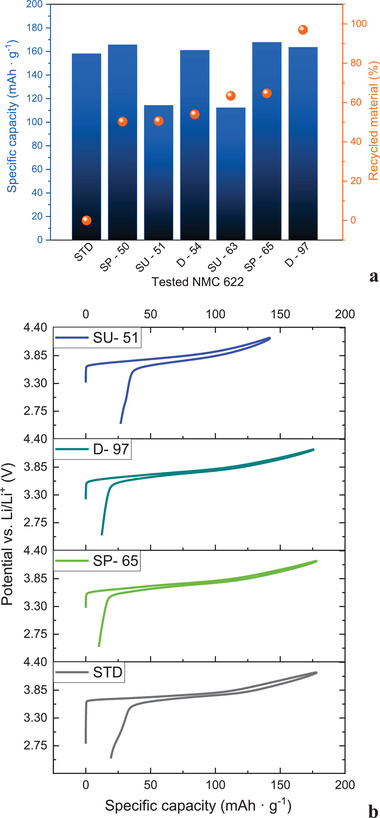
a) Recorded capacity values for the first cycles of the tested synthesized NMC 622 materials with the corresponding recycled material content (%), b) representative first formation cycles for the selected materials.

**Figure** **7**a presents the capacity retention at the tested current densities for the tested NMC 622 synthesized materials compared with the commercially available one. A lower capacity recorded for cells containing unpurified active material both SU‐63% and SU‐51% can be distinguished. The cells comprising of the material from disassembly and purified shredding processes exhibit similar capacity retention at 0.32 A g^−1^. With an increase of the current density, there can be noted higher capacity retention for the cells comprising of SP‐65% and SP‐50%.

The highest capacity retention of 74% was measured for SP‐65% at the highest current density of 1.6 A g^−1^, whereas 66% of the recovered capacity was recorded for the standard NMC 622 as well as for the cells testing D‐97%.

Figure 7b shows the cycle life performance with a corresponding protocol applied, Figure S5a in the Supporting Information, and differential capacity profiles (c) for the cathode half‐cells. It can be noted from Figure 7b that the highest capacities were observed for the cell containing SP‐65% of recycled material. Similarly, D‐97% exhibited similar capacity values as the tested commercially available NMC 622. Whereas, materials from the unpurified streams: SU‐51% and 63% demonstrated ≈60 mAh g^−1^ lower capacity than standard NMC 622.

It can be also noted that the discharge profiles have no visible resistive behavior for the recycled materials when compared to the pristine NMC 622, see Figure S5b in the Supporting Information.

From Figure S5c in the Supporting Information, a decrease of the internal resistance can be observed for the discharge cycles in all cells, highlighted in red and green colors, for the 10th and 40th cycles. The variation in the differential capacity profiles at the beginning of the discharge cycles can indicate a faster kinetics for Li^+^ insertion.^[^ ^45^ ^]^

## 3 Energy Comparison of the Disassembly and Shredding Processes

**Figure 8:**
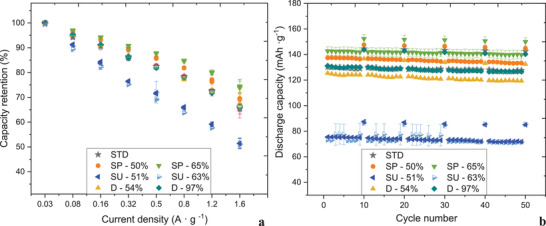
a) Capacity retention versus applied current density, b) cycle life performance of the tested NMC 622 materials.

**Figure** **8**a demonstrates relation between measured impurity level of Cu and Al measured by ICP‐OES versus capacity ratio for the tested NMC 622 of the materials synthesized from the shredding processes. Figure 8b refers to the energy demand for electricity per cell, where it is compared with the noted gravimetric energy. The highest energy demand for electricity (Wh cell^−1^) and noted energy (Wh g^−1^) was calculated for SP‐65%. However, much lower energy demand for electricity (358.3 Wh cell^−1^) and only 50 mWh g^−1^ lower measured energy is noted for the disassembly process.

**Figure 9:**
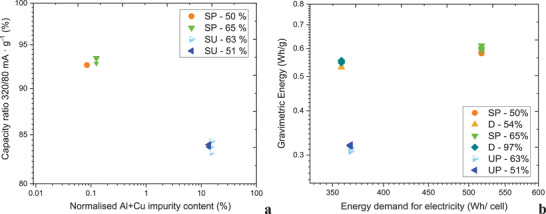
a) Capacity ratio 320/80 mA g^−1^ (%) versus normalized Al/Cu impurity level (%), b) measured gravimetric energy measured from the discharge (320 mA g^−1^) versus energy demand for electricity (Wh cell^−1^).

In addition to this, the Sankey diagrams, **Figure** **9**, illustrate each of the process: disassembly, shredded with and without purification incorporated that contribute to the total energy demand for electricity per cell. It is clear that purification process, applied to remove the current collector impurities in the shredded stream, increases significantly the overall electricity demand. The disassembly process has demonstrated the lowest energy demand for electricity per cell, whereas, the highest energy demand is required for the shredded process with applied purification step.

## 4 Conclusions

This work demonstrates a recycling scheme for Nissan Gen 1 cells containing LMO/NCA cathodes, to produce Ni‐rich cathode materials using physical disassembly and shredding routes. Cathodic black mass from disassembled cathode electrodes is produced using a low power delamination process. Delamination was achieved using a weak acid at lab scale with low power ultrasound and pilot scale with stirring. A peeling efficiency of 99% with some presence of Li and Al in the leaching solutions was observed. A high purity cathodic black mass was produced with LMO‐NCA, carbon black, and polyvinylidene fluoride (PVDF). The black mass after shredding was concentrated using size separation, and contained significantly more aluminium and copper current collectors, along with graphite, carbon black, and PVDF.

The purity of the transition metal containing black mass was higher from the disassembly route compared to shredding and sorting. Transitional metals were extracted through a hydrometallurgical process, in which soluble salts were concentrated into a solution. This step produced a waste by‐product of graphite, PVDF, and carbon black (shredded black mass), and PVDF‐carbon black (disassembled cathode black mass), the transition metal solutions were further purified, and lithium, copper, and aluminium were removed through sequential salt precipitation steps. The greater the level of impurity, the more precipitation steps were required. The final transition metal salt solution exhibited a lower concentration of impurities from disassembly compared to the shredded black mass.

Upcycled NMC 622 cathode was manufactured from this reclaimed cathodic black mass and compared to that manufactured from pure as‐received salts from the manufacturer. The level of cobalt, nickel, and manganese in the solution was altered to the correct ratio for NMC 622, and then manufactured using a co‐precipitation and subsequent lithiation step. The effect of the impurity levels was investigated. Significant levels of copper and aluminium impurities from the as‐received purified shredded black mass had a detrimental effect upon the observed specific capacity of the NMC 622, however after purification and with lower levels of impurities, the performance of NMC 622 was similar to the samples made with less and no recycled transition metals (168 and 164 mAh g^−1^ for SP‐65% from shredded and D‐97% from disassembled reclaimed transition metals, respectively).

The techno‐economic evaluation shows that the economics of the process is highly dependent upon the cathode purification step (impurity removal from the transition metal salt solution) and the heat treatment of the NMC 622. If more impurities are present, the energy input will increase due to the repeated purification steps required. However, if the input from the shredded black mass can be further purified through physical processing methods, the impurity levels can be lowered, and this may reduce the cathode purification step energy input. In comparison, the disassembly route is currently time‐consuming and labor‐intensive to do, particularly if the cells are damaged. Further work is needed to automate and speed up the disassembly route, and despite the higher purity waste streams, the benefit of this versus the cost and energy for disassembly may outweigh the benefit.

There are benefits to both disassembly and shredding and sorting routes to recycling for LIBs. Shredding can deal with any cell, whether damaged, scrap, or EOL—however the purity of the waste streams is low in comparison to that from a disassembly process. The disassembly process can disassemble only undamaged cells. Cathodic black mass feed stocks can be used from either process to manufacture and upscale to other transition metal containing oxides such as NMC 622.

## 5 Experimental Section

### Cathodic Black Mass Recovery

*Disassembly Process*: Two methods for reclaiming the cathode black mass were investigated, the first was based upon a disassembly method which has been described previously.^[^ ^30^ ^]^ Here an EOL Nissan pouch cell containing 25NCA:75LMO cathode was opened with a ceramic blade in a fume hood, and the anode and cathodes were individually removed for further black mass separation. The coated aluminium electrode was then further separated using a heated solution of oxalic acid (0.5 m at 50 °C) for 5 min with the use of ultrasonic bath. The separated foil and cathode black mass were subsequently dried in a convection oven at 60 °C for 2 h.

*Shredding Process*: The second method for separation and transition metal concentration was achieved via a shredding process. The cells (containing NMC‐rich cathode material from an industrial automotive application) were processed using a single‐shaft knife mill shredder, then separated using a combination of delamination, screening, and density separation. The black mass was recovered as a wet filter cake for further processing.

**Figure 10:**
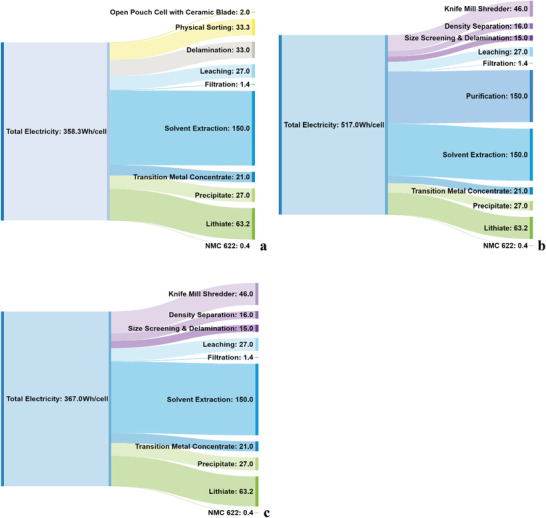
The Sankey diagrams of energy demand for electricity by process in Wh cell^−1^, illustrating processes: a) disassembly, b) shredded with purification, and c) shredded without purification.

*Transition Metal Leaching*: The cathodic black mass was treated using hydrometallurgical methods, presented in **Figure** **10**. Cathodic black mass was slurried in water at ≈40% w/w, then a stoichiometric +10% amount of sulfuric acid (95% w/w) was added slowly with excess (indicated by cessation of effervescence) hydrogen peroxide (30% w/w) in order to solubilize the transition metals and metallic current collectors. Any insoluble material was filtered off and retained for further analysis.

*Purification Process*: It was found that black mass from simple shredded waste processing contained a much higher concentration of Cu and Al compared to the disassembly waste process (by a factor of ≈20). For this source of black mass, a preliminary separation was made by the selective precipitation of the respective hydroxides by addition of 46% sodium hydroxide to a pH of 3.0. The precipitate was filtered off leaving a solution comparable to that produced directly from the disassembly waste process.

*Solvent Extraction*: The filtered, solubilized metal solution then underwent a liquid–liquid extraction process for separation of Al and Cu. The process was optimized by repeating experiments with adjustment of the extraction pH until the desired purity of solution was obtained, as measured using ICP‐OES. The choice of extractant and conditions used for this separation are proprietary to ICoNiChem. The pure solution was precipitated to produce NMC mixed metal hydroxide using 47% sodium hydroxide and washed to separate Na and Li.

*Transition Metal Concentrate*: The resulting precipitate was redissolved in sulfuric acid and topped up with the required amounts of virgin nickel, cobalt, and manganese sulfate salts to produce a Ni:Mn:Co concentration of 6:2:2 . This material was then sent to the project partner TFP Hydrogen for onward lithium‐ion cathode manufacture.

*NMC 622 Manufacture: Precipitation*: The transition metal solution (Ni:Mn:Co 6:2:2, 2M) was heated to 60 °C with stirring. A base solution of ammonium hydroxide and sodium hydroxide was prepared, with a ratio of 1:1, and added dropwise to the metal solution, aiding the formation of metal hydroxide precipitate, until pH 11 was reached and stabilized. The mixture was continuously stirred without heating overnight. The resulting brown metal hydroxide was then washed with filtration and the resulting filter cake was dried in an oven overnight at 100 °C.

*Lithiation*: The dry NMC hydroxide was milled with lithium hydroxide, with 5% excess of the overall NMC molarity. The lithiated hydroxide powder was then heated in a tube furnace under 21% O_2_/Ar at 850 °C for 6 h. The as‐made cathode materials were then sieved to less than 45 µm in size under an inert atmosphere. XRD was performed on the samples to confirm crystal structure as well as particle size.

### Materials Characterization

To obtain samples for ICP‐OES, the cathodic black mass was dissolved in hot aqua regia. The resulting solution was diluted to appropriate volumes and the metals contained were analyzed by ICP‐OES. XRD was performed with the use of an X‐ray Proto diffractometer with the scan range of 2θ from 15° to 70° with a step size of 12 s per step and a scan speed of 2 per second. The lattice constants were calculated with the use of GSAS software.^[^ ^46^, ^47^ ^]^ An SEM, Jeol 7000, equipped with the EDS detector was performed with the use of microscope at the accelerated voltage of 15.0 kV and the working distance of 6.0 mm used for the image acquisition and 10.0 mm for the EDS mapping, respectively.

Cross‐sections of the calendared coatings obtained from the synthesized and commercial materials were prepared. Samples were polished with the use of Argon Ion Milling Machine (Hitachi) with the milling conditions: accelerated voltage of 5 kV, ion beam current of ≈130 µA, milling time 3 h.

Next, the polished samples were analyzed with SEM (Philips XL‐30 FEG ESEM) equipped with EDS detector at accelerated voltage of 20 kV, WD of ≈11 mm (optimal conditions for EDS for the system).

### Electrochemical Testing and Characterization

The cathode slurries were prepared with the use of a Thinky ARE 250 dual asymmetric planetary mixer (Thinky, USA). A binder solution (8 wt% of PVDF Solef 5130 in NMP), conductive additive C65 (Timcal) together with an additional 50% NMP was placed in the pot and mixed first at 500 rpm for 1 min followed by 2000 rpm for 5 min (without a break between the two settings). Subsequently, the active material NMC 622 and the remaining solvent were added and the same stirring conditions were applied, followed by the degassing step at a speed of 2200 rpm for 3 min.

As‐prepared slurries were coated onto the aluminium foil using a draw‐down coater (K Paint Applicator, RK Printcoat Instruments, UK) with a doctor blade. The prepared coatings were dried on a hot plate at 80 °C and then transferred to the vacuum oven and dried at 120 °C for ≈24 h prior to the calendaring and cell construction. The coated foils were subsequently calendared at 80 °C to 35% porosity with the use of MTI calendar.

The cathode coatings were tested electrochemically in coin cell half‐cells. The coat weight of the electrodes was adjusted to achieve areal capacity of ≈1 mAh cm^−2^. Dried coatings were processed in a dry room with a dew point of −45 °C. 2032 coin cells were constructed with positive electrode disks (14.8 mm in diameter), tri‐layer 2025 separator (Celgard), and lithium metal disk acting as a counter electrodes (15.0 mm, 150 µm thick) filled with 70 µm of 1 mol L^−1^ PuriEL Battery Electrolyte (R&D281) from Soulbrain (Michigan, USA). The composition of the electrolyte was 1.0 m LiPF_6_ in EC/EMC (ethylene carbonate/ethyl methyl carbonate) 3/7 v/v +2% wt. VC (vinylene carbonate) electrolyte and sealed with a hydraulic crimper (MSK‐110, MTI Corporation, USA).

Electrochemical testing was performed on a Bio‐Logic BCS 805 series cycler. Initial capacities and formation were obtained by applying the following protocol: 10 mA g^−1^ charge to 4.2 V versus Li/Li+ and delithiation to 2.5 V versus Li/Li+ at the same current density with the same step repeated twice. Current density testing was performed by applying three cycles per 30, 80, 160, 320, 500, 800, 1200 and 1600 mA g^−1^. Followed by cycling by setting 80 mA g^−1^ at the charge and 320 mA g^−1^ for the discharge cycles, respectively, repeated every 10th cycle for total of 50 cycles for the test.

## Conflict of Interest

The following people are employees of the following companies: C.M., N.vD; TFP hydrogen products who synthesized the NMC 622 cathode, S.H., RSBruce, who developed the shredding and sorting process, P.C., E.C.; ICoNiChem, who reclaimed the transition metal salts, M.C. and R.S. MSolv, who performed the LCA and TE analysis, and provided the laser opening device.

## Supporting information

Supporting Information

## Data Availability

The data that support the findings of this study are available from the corresponding author upon reasonable request.

## References

[1] B. Scrosati , J. Solid State Electrochem. 2011, 15, 1623.

[2] M. V. Reddy , A. Mauger , C. M. Julien , A. Paolella , K. Zaghib , Materials 2020, 13, 1884. 32316390 10.3390/ma13081884 PMC7215417

[3] K. Brandt , Solid State Ionics 1994, 69, 173.

[4] T. Or , S. W. D. Gourley , K. Kaliyappan , A. Yu , Z. Chen , Carbon Energy 2020, 2, 6.

[5] Y. Ding , Z. P. Cano , A. Yu , J. Lu , Z. Chen , Electrochem. Energy Rev. 2019, 2, 1.

[6] L. Gaines , Sustainable Mater. Technol. 2018, 17, e00068.

[7] O. Velázquez‐Martínez , J. Valio , A. Santasalo‐Aarnio , M. Reuter , R. Serna‐Guerrero , Batteries 2019, 5, 68.

[8] J.‐M. Tarascon , M. Armand , Nature 2001, 414, 359. 11713543 10.1038/35104644

[9] T. Waldmann , A. Iturrondobeitia , M. Kasper , N. Ghanbari , F. Aguesse , E. Bekaert , L. Daniel , S. Genies , I. J. Gordon , M. W. Löble , E. De Vito , M. Wohlfahrt‐Mehrens , J. Electrochem. Soc. 2016, 163, A2149.

[10] Y. Kobayashi , T. Kobayashi , K. Shono , Y. Ohno , Y. Mita , H. Miyashiro , J. Electrochem. Soc. 2013, 160, A1181.

[11] T. Langner , T. Sieber , J. Acker , Sci. Rep. 2021, 11, 6316. 33737549 10.1038/s41598-021-85575-x PMC7973563

[12] C. Fear , D. Juarez‐Robles , J. A. Jeevarajan , P. P. Mukherjee , J. Electrochem. Soc. 2018, 165, A1639.

[13] D. Aurbach , B. Markovsky , A. Rodkin , M. Cojocaru , E. Levi , H.‐J. Kim , Electrochim. Acta 2002, 47, 1899.

[14] G. A. Blengini , et al., European Commission, Study on the EU's list of Critical Raw Materials – Final Report (2020), 2020.

[15] A. Sonoc , J. Jeswiet , V.i K. Soo , Procedia CIRP 2015, 29, 752.

[16] M. Chen , X. Ma , B. Chen , R. Arsenault , P. Karlson , N. Simon , Y. Wang , Joule 2019, 3, 2622.

[17] X. Zeng , J. Li , N. Singh , Crit. Rev. Environ. Sci. Technol. 2014, 44, 1129.

[18] R. Sommerville , P. Zhu , M. A. Rajaeifar , O. Heidrich , V. Goodship , E. Kendrick , Resour., Conserv. Recycl. 2020, 165, 105219.

[19] R. Zhan , Z. Oldenburg , L. Pan , Sustainable Mater. Technol. 2018, 17, e00062.

[20] G. Zhang , Z. Du , Y. He , H. Wang , W. Xie , T. Zhang , Sustainability 2019, 11, 2363.

[21] R. Zhan , T. Payne , T. Leftwich , K. Perrine , L. Pan , Waste Manage. 2020, 105, 39. 10.1016/j.wasman.2020.01.035 32018141

[22] W. Gao , X. Zhang , X. Zheng , X. Lin , H. Cao , Y.i Zhang , Z. Sun , Environ. Sci. Technol. 2017, 51, 1662. 28081362 10.1021/acs.est.6b03320

[23] R. Zheng , W. Wang , Y. Dai , Q. Ma , Y. Liu , D. Mu , R. Li , J. Ren , C. Dai , Green Energy Environ. 2017, 2, 42.

[24] Y. Yang , G. Huang , S. Xu , Y. He , X. Liu , Hydrometallurgy 2016, 165, 390.

[25] T. Nshizirungu , M. Rana , Y. T. Jo , J.‐H. Park , J. Hazard. Mater. 2021, 414, 125575. 34030417 10.1016/j.jhazmat.2021.125575

[26] Y. Chen , N. Liu , F. Hu , L. Ye , Y. Xi , S. Yang , Waste Manage. 2018, 75, 469. 10.1016/j.wasman.2018.02.024 29478957

[27] O. Dolotko , I. Z. Hlova , Y. Mudryk , S. Gupta , V. P. Balema , J. Alloys Compd. 2020, 824, 153876.

[28] J. Xiao , J. Li , Z. Xu , J. Hazard. Mater. 2017, 338, 124. 28544937 10.1016/j.jhazmat.2017.05.024

[29] M.‐M. Wang , C.‐C. Zhang , F.‐S. Zhang , Waste Manage. 2016, 51, 239. 10.1016/j.wasman.2016.03.006 26965214

[30] J. Marshall , D. Gastol , R. Sommerville , B. Middleton , V. Goodship , E. Kendrick , Metals 2020, 10, 773.

[31] C. Herrmann , A. Raatz , M. Mennenga , J. Schmitt , S. Andrew , in Leveraging Technology for a Sustainable World, (Eds: D. Dornfeld , B. Linke ), Springer, Berlin, Heidelberg 2012, pp. 149–154.

[32] L. Li , P. Zheng , T. Yang , R. Sturges , M. W. Ellis , Z. Li , JOM 2019, 71, 4457.

[33] E. Mossali , N. Picone , L. Gentilini , O. Rodrìguez , J. M. Pérez , M. Colledani , J. Environ. Manage. 2020, 264, 110500. 32250918 10.1016/j.jenvman.2020.110500

[34] G. Harper , R. Sommerville , E. Kendrick , L. Driscoll , P. Slater , R. Stolkin , A. Walton , P. Christensen , O. Heidrich , S. Lambert , A. Abbott , K. Ryder , L. Gaines , P. Anderson , Nature 2019, 575, 75. 31695206 10.1038/s41586-019-1682-5

[35] M. A. Rajaeifar , M. Raugei , B. Steubing , A. Hartwell , P. A. Anderson , O. Heidrich , J. Ind. Ecol. 2021, 25, 1560.

[36] A. Chagnes , B. Pospiech , J. Chem. Technol. Biotechnol. 2013, 88, 1191.

[37] M. Chen , Z. Zheng , Q. Wang , Y. Zhang , X. Ma , C. Shen , D. Xu , J. Liu , Y. Liu , P. Gionet , I. O'connor , L. Pinnell , J. Wang , E. Gratz , R. Arsenault , Y. Wang , Sci Rep. 2019, 9, 1654. 30733518 10.1038/s41598-018-38238-3 PMC6367435

[38] Y. He , X. Yuan , G. Zhang , H. Wang , T. Zhang , W. Xie , L. Li , Sci. Total Environ. 2021, 766, 142382. 33183828 10.1016/j.scitotenv.2020.142382

[39] S. Yang , X. Wang , X. Yang , Y. Bai , Z. Liu , H. Shu , Q. Wei , Electrochim. Acta 2012, 66, 88.

[40] Y. Xia , J. Zheng , C. Wang , M. Gu , Nano Energy 2018, 49, 434.

[41] Q. Sa , J. A. Heelan , Y. Lu , D. Apelian , Y. Wang , ACS Appl. Mater. Interfaces 2015, 7, 20585. 26325672 10.1021/acsami.5b04426

[42] H. Li , J. Li , X. Ma , J. R. Dahn , J. Electrochem. Soc. 2018, 165, A1038.

[43] P. Y. Liao , J. G. Duh , S. R. Sheen , J. Electrochem. Soc. 2005, 152, A1695.

[44] K. R. Tallman , G. P. Wheeler , C. J. Kern , E. Stavitski , X. Tong , K. J. Takeuchi , A. C. Marschilok , D. C. Bock , E. S. Takeuchi , J. Phys. Chem. C 2021, 125, 58.

[45] H. Gao , X. Zeng , Y. Hu , V. Tileli , L. Li , Y. Ren , X. Meng , F. Maglia , P. Lamp , S.‐J. Kim , K. Amine , Z. Chen , ACS Appl. Energy Mater. 2018, 1, 2254.

[46] A. C. Larson , R. B. Von Dreele , General Structure Analysis System (GSAS), Los Alamos Natl. Lab. Rep. LAUR 86–748, 2000.

[47] B. H. Toby , J. Appl. Crystallogr. 2001, 34, 210.

