# Supplementary material for: Reclaimed and Up‐Cycled Cathodes for Lithium‐Ion Batteries
Source: Glob Chall. 2022 Jun 9;6(12):2200046. doi: 10.1002/gch2.202200046 (PMC9749079; doi:10.1002/gch2.202200046)
Supplement: Supplementary file 1 — Supporting Information [file GCH2-6-2200046-s001.pdf]

Global Challenges
Open Access

Supporting Information

for *Global Challenges,* DOI: 10.1002/gch2.202200046

Reclaimed and Up-Cycled Cathodes for Lithium-Ion Batteries

Dominika Gastol, Jean Marshall, Elizabeth Cooper, Claire Mitchell, David Burnett, Tengfei Song, Roberto Sommerville, Bethany Middleton, Mickey Crozier, Robert Smith, Sam Haig, Con Robert McElroy, Nickvan Dijk, Paul Croft, Vannessa Goodship, and Emma Kendrick**

Reclaimed and up-cycled cathodes for Lithium-ion batteries

Dominika Gastol[1]*, Jean Marshall[2], Elizabeth Cooper[3], Claire Mitchell[4], David Burnett[1], Tengfei Song[1], Roberto Sommerville[1], Bethany Middleton[2], Mickey Crozier [6], Robert Smith [6], Sam Haig[5], Con Robert McElroy[7], Nick van Dijk[4], Paul Croft[3], Vannessa Goodship[2], Emma Kendrick[1]*

[1] School of Metallurgy and Materials, University of Birmingham, Birmingham B15 2TT, UK2

[2] WMG, University of Warwick, Coventry CV4 7AL, UK

[3] ICoNiChem, Widnes Ltd, Moss Bank Road, Widnes, Cheshire WA8 0RU, United Kingdom

[4] TFP Hydrogen Products, Units 5 & 6, Merchants Quay, Pennygillam Industrial Estate, Launceston, Cornwall, PL15 7QA, UK

[5]RSBruce metals and Machinery Ltd, March Street, Sheffield, South Yorkshire, S9 5DQ, UK

[6]MSolv, Oxonian Park, Langford Locks, Kidlington, Oxford OX5 1FP, UK

[7]Green Chemistry Centre of Excellence, Department of Chemistry, University of York, Heslington, York, YO10 5DD

e-mail: D.A.Gastol@bham.ac.uk

E.Kendrick@bham.ac.uk

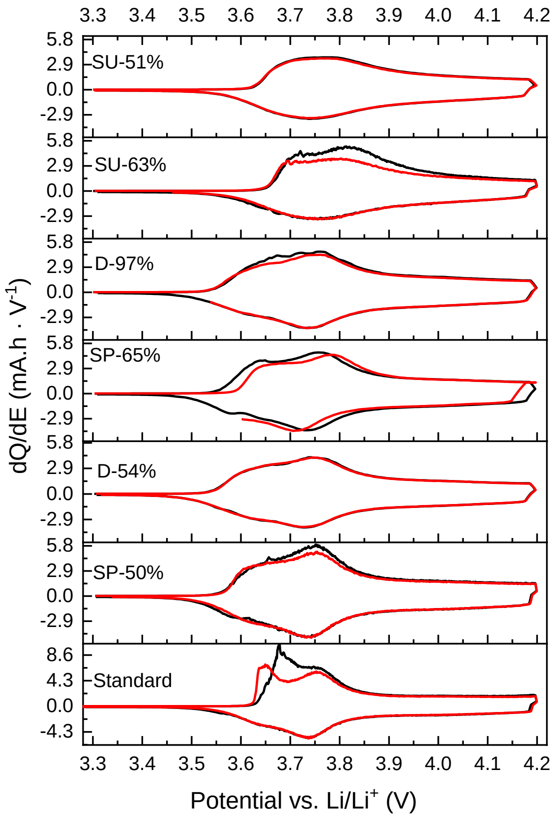

Figure S1 Differential Capacity (dQ/dV) of two first formation cycles for the tested NMC 622.

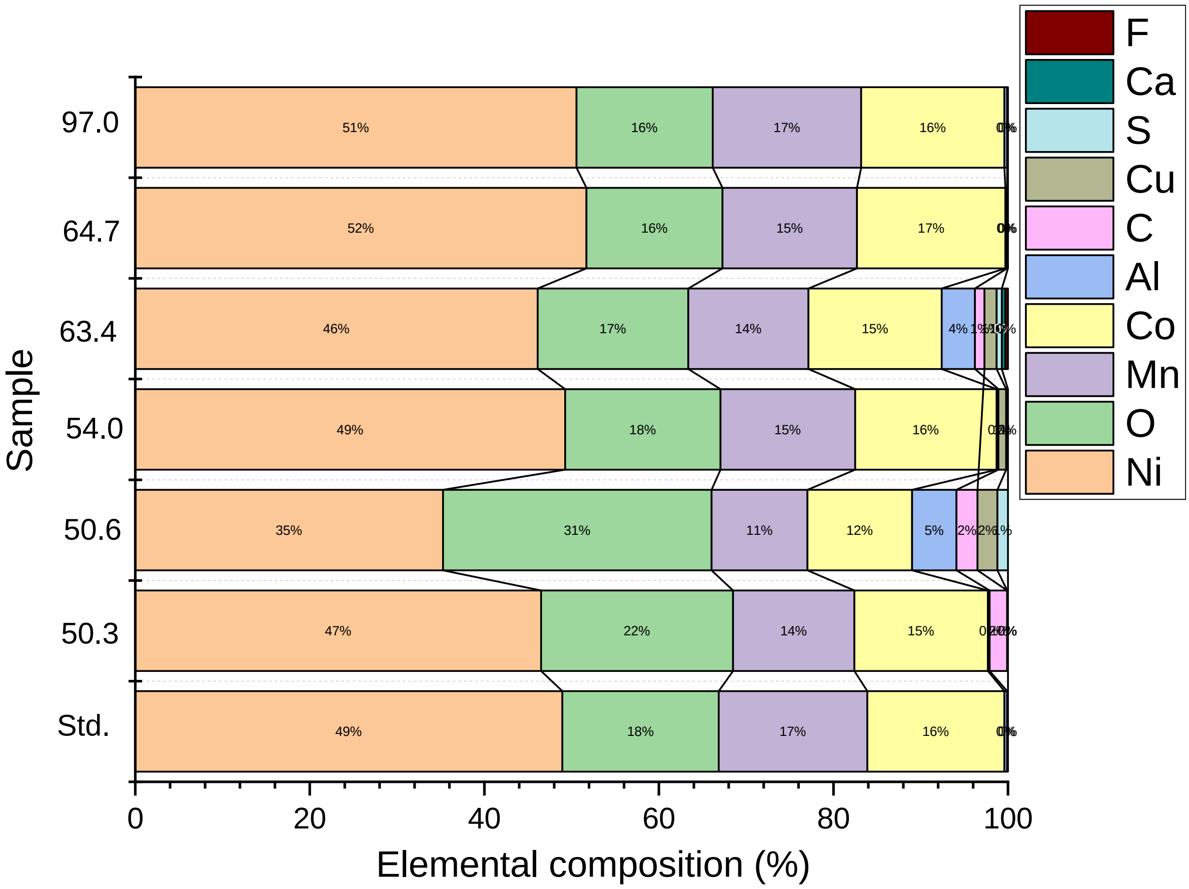

Figure S2 Elemental composition from the EDS analysis of the synthesised NMC622 powders.

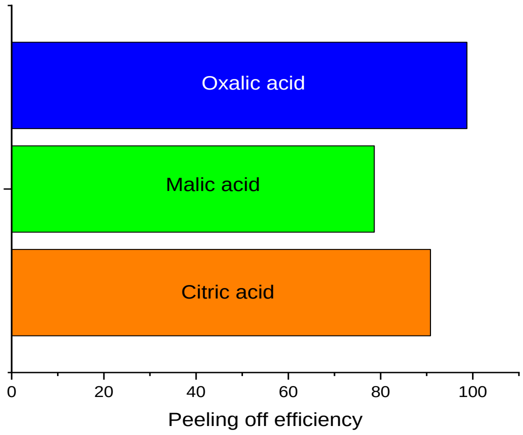

Figure S3 Peeling off efficiency of the organic acids applied for separation of the cathodic black mass from Al current collectors in the Disassembly Process.

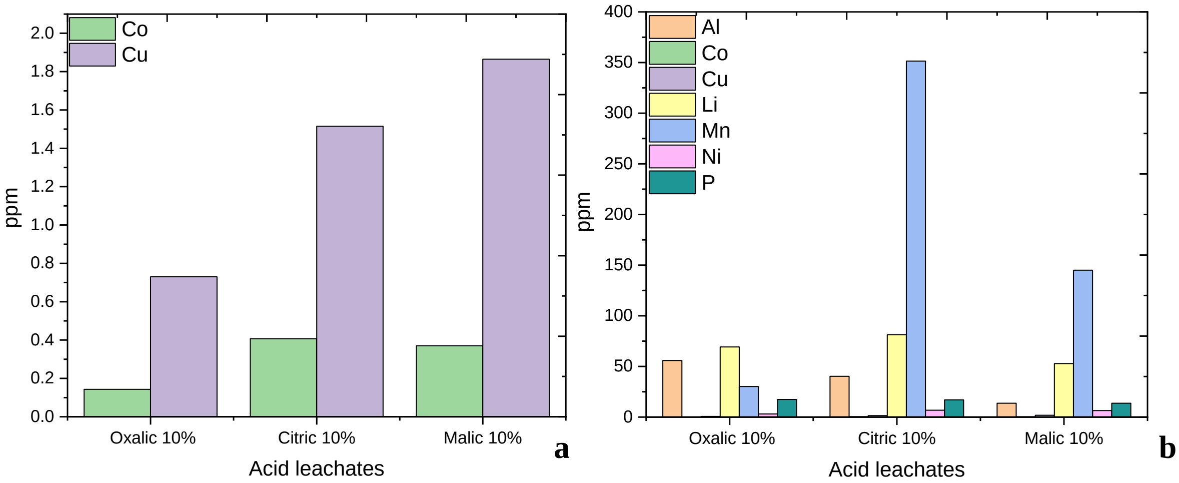

Figure S4. ICP-OES results of the acid leachates (10%) after ultrasonic separation used in a disassembly process.

Table S1. ICP-OES analysis of wastewater (WWS) obtained from the shredded material processing.

PPM	WWS
**Al**	0.1
**Co**	0.5
**Cu**	20.7
**Li**	524.9
**Mn**	3.2
**Ni**	0.6
**P**	961.6

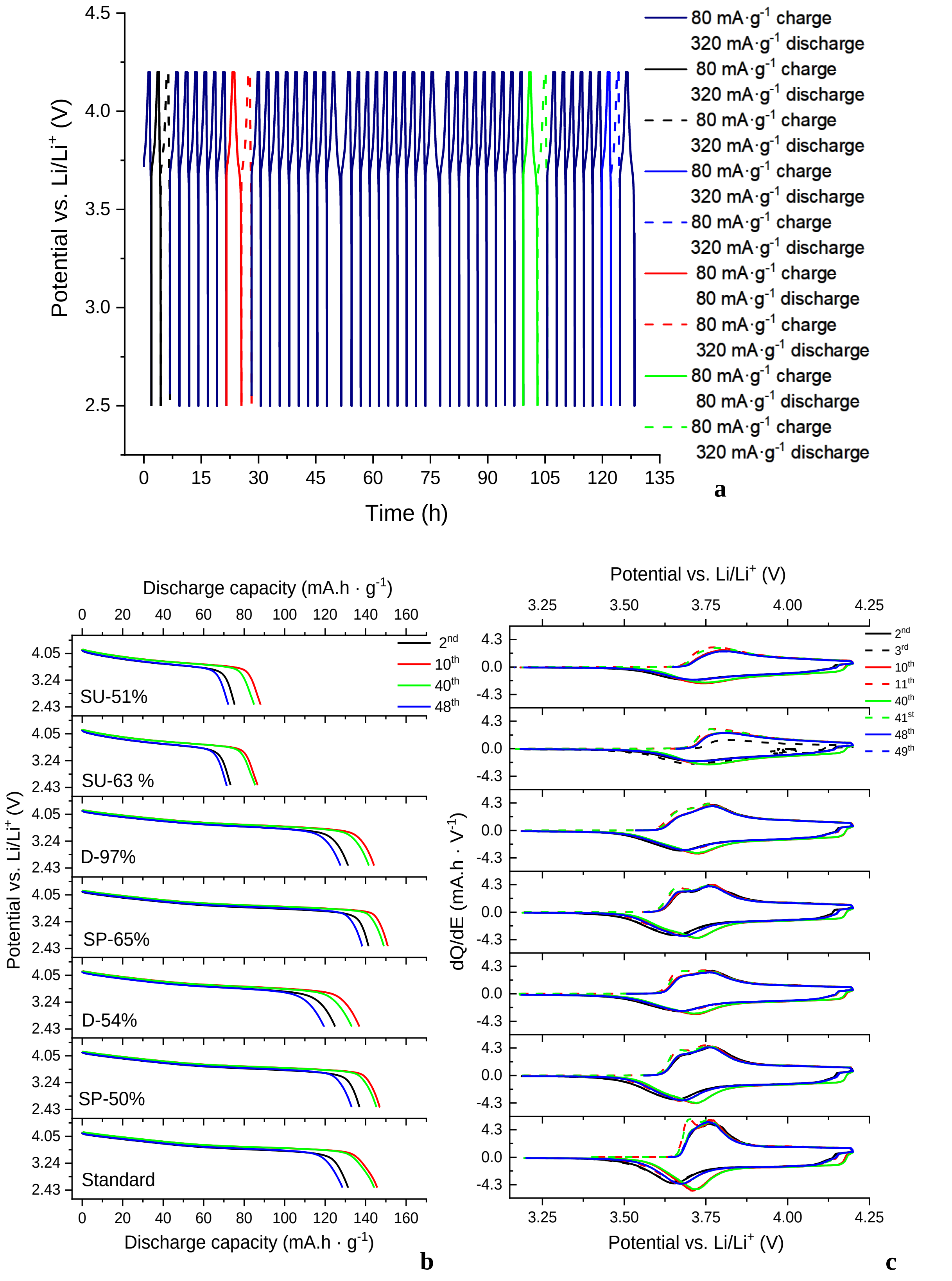

Figure S5. (a) Applied cycle life protocol (b) with representative discharge profiles and corresponding (c) Differential Capacity (dQ/dV) from the cycle life performance.

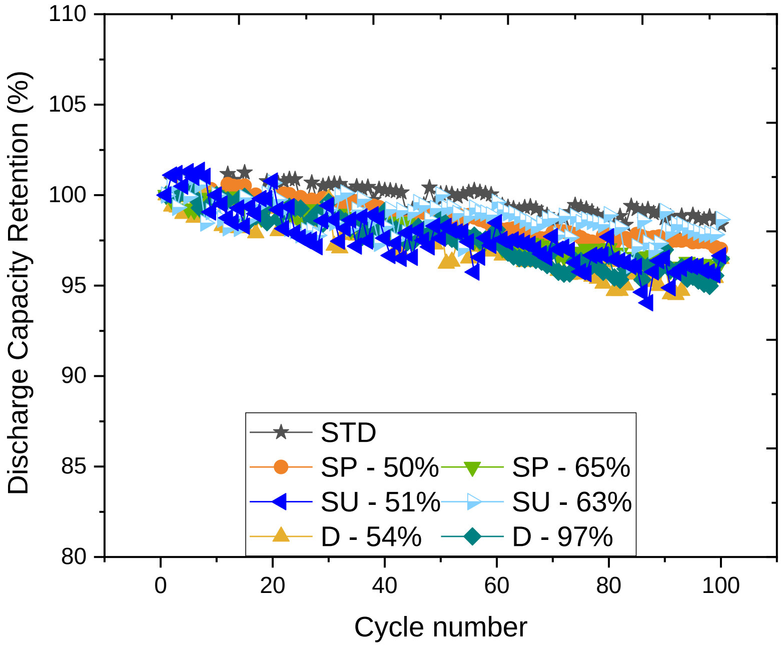

Figure S6 Cycle life performance of the tested cathode half-cells comprising of the tested NMC 622 materials, presented as discharge capacity retention (%) vs. Cycle number.

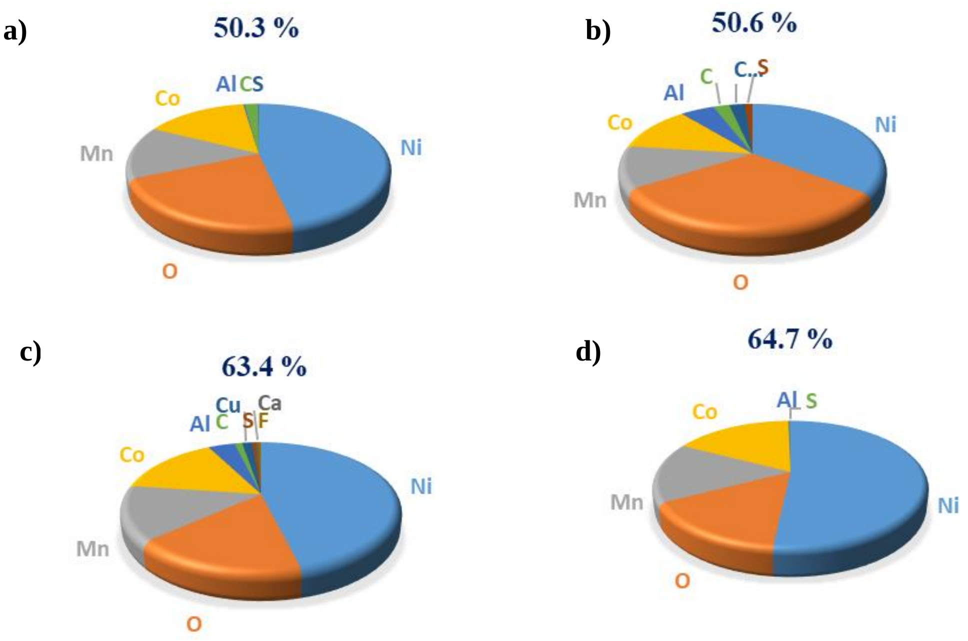

Figure S7. EDS analysis of the NMC 622 powders obtained from the shredding process: SU: a), c), SP: c), d).

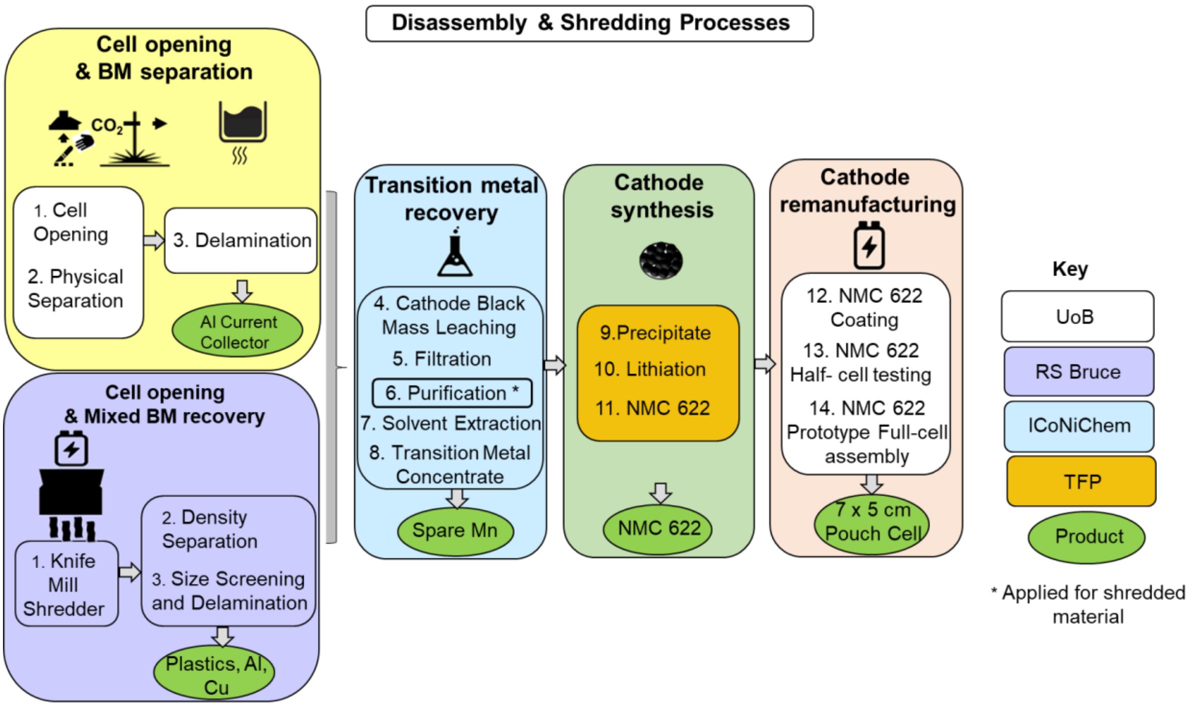

Figure S8. Schematic of the disassembly processes, top and bottom diagrams, respectively, demonstrated in this research work. The disassembly route comprises of 25 process units that start from the cell preparation, opening, disassembly, transition metal recovery steps, through cathode NMC 622 material manufacturing by a co-precipitation method and lastly battery re-manufacturing with the use of synthesised material from the recycling stream. Each of the process steps represented by the project partners and an associated activity result in the product outcome. The process waste stream has not been included in this diagram. However, the mitigation of the waste generation by analysing alternative activities and energy sources have been considered throughout the project.

Cross-sectioned electrode coatings with a corresponding aluminium and copper distribution in the selected areas have been illustrated in the Figure S9. The quantitative results have been summaries in the Table S2.

It can be observed that Al has been detected in all investigated samples, with the lowest wt.% content noted for SP-50%, D-97%, SP-65%, and STD. The highest wt.% was measured for SU-51% and SU-63%. Copper was detected only in unpurified samples SU-51% and 63%.

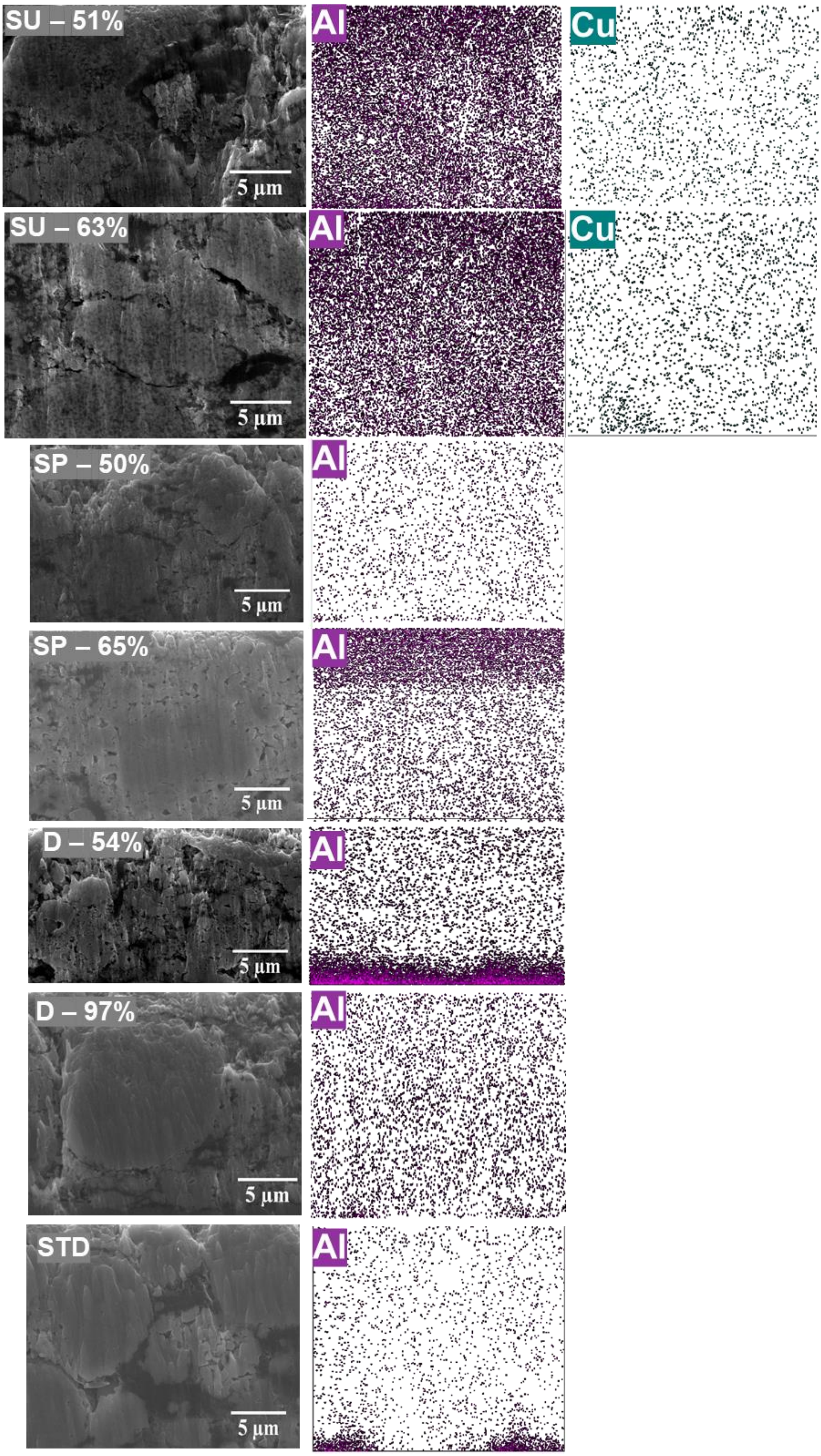

Figure S9 Cross-section micrographs of the calendared electrodes obtained from the synthesised and commercial NMC 622 materials with the corresponding distribution of Al and Cu obtained from the EDS scans.

Table S2. EDS analysis of the cross-sectioned coatings, comprising of the investigated NMC 622 materials, indicating Al and Cu weight %.

Coating ID	Weight %	
	Al	Cu
SU – 51%	4.34 ± 0.06	0.70± 0.09
SU – 63%	4.19 ± 0.06	0.88 ± 0.09
SP – 50%	0.06 ± 0.04	-
SP – 65%	0.97 ± 0.03	-
D- 54%	2.33 ± 0.05	-
D- 97%	0.10 ± 0.03	-
STD	0.70± 0.05	-

The Table S3, summarises the comparison between the reported specific capacities from the first cycle (mAh/g), efficiencies (%)obtained, electrode loading (mg/cm^2), operating voltage and applied current density of re-synthesised NMC 622 cathode material recovered from different recycling streams.

Table S3 Reported tested NMC 622 obtained from different recycling streams.

Work reported	**Capacity from formation cycle (mAh/g)**	**First cycle efficiency (%)**	**Electrode coat weight (mg/cm^2**	**Operating voltage vs. Li/Li$^+$(V)**	**Applied current density**	**Comments**	**Reference**
**upNMC 622**	162.9 ±0.2	83.4 ±0.8	7.0	3 – 4.3	20 mA/g	Active material obtained from chemically delithiated NMC 111	[1]
**SP-65%**	167.7 ± 0.8	94.1 ±0.2	10.5 ±0.4	2.5 – 4.2	10 mA/g	Active material obtained from EoL Nissan Leaf pouch cells	Our work
**SP-50%**	165.6 ±0.6	93.1 ±0.2					
**D-97%**	163.5±0.3	93.1 ±0.1					
**P-NMC**	173.1	88.8	20	2.5 – 4.3	0.1 C	Applied Al_2O_3 coating to synthesised NMC622, samples: D and W	[2]
**D-NMC**	172.4	88.5					
**W-NMC**	178.5	91.2					

References:

[1] T. Wang *et al.*, "Flux upcycling of spent NMC 111 to nickel-rich NMC cathodes in reciprocal ternary molten salts," *iScience*, vol. 25, no. 2, p. 103801, 2022, doi: 10.1016/j.isci.2022.103801.

[2] B. Chen *et al.*, "Systematic Comparison of Al3+ Modified LiNi0.6Mn0.2Co0.2O2 Cathode Material from Recycling Process," *ACS Appl. Energy Mater.*, vol. 2, no. 12, pp. 8818–8825, 2019, doi: 10.1021/acsaem.9b01814.
